# Supplementary material for: Genome sequencing and functional characterization of a Dictyopanus pusillus fungal enzymatic extract offers a promising alternative for lignocellulose pretreatment of oil palm residues
Source: PLoS One. 2020 Jul 30;15(7):e0227529. doi: 10.1371/journal.pone.0227529 (PMC7392265; doi:10.1371/journal.pone.0227529)
Supplement: S1 File — (DOCX) [file pone.0227529.s001.docx]

**Genome sequencing and functional characterization of a *Dictyopanus pusillus* fungal enzymatic extract offers a promising alternative for lignocellulose pretreatment of oil palm residues**

Andrés M. Rueda^1, 2, 3^, Yossef López de los Santos^1^, Antony T. Vincent^1^, Myriam Létourneau^1^, Inés Hernández^3^, Clara I. Sánchez^3, 4^, Daniel Molina V. ^5^, Sonia A. Ospina^2^, Frédéric J. Veyrier^1^ & Nicolas Doucet^1, 6, *^

^1^Centre Armand-Frappier Santé Biotechnologie, Institut national de la recherche scientifique (INRS), Université du Québec, Laval, Canada. ^2^Instituto de Biotecnología, Universidad Nacional de Colombia, Bogotá, Colombia. ^3^Centro de Estudios e Investigaciones Ambientales, Universidad Industrial de Santander, Bucaramanga, Colombia. ^4^Escuela de Microbiología, Universidad Industrial de Santander, Bucaramanga, Colombia. ^5^Escuela de Química, Universidad Industrial de Santander, Bucaramanga, Colombia. ^6^PROTEO, Québec Network for Research on Protein Function, Engineering, and Applications, Québec, Canada.

*To whom correspondence should be addressed: Nicolas Doucet, Centre Armand-Frappier Santé Biotechnologie, Institut national de la recherche scientifique (INRS), Université du Québec, 531 Boulevard des Prairies, Laval, QC, Canada, H7V 1B7; [nicolas.doucet@inrs.ca](mailto:nicolas.doucet@inrs.ca).

**Table S1.** Putative laccases identified in the genome of *Dictyopanus pusillus* LMB4.

| Locus tag | Length a.a. | Number of similar laccases | multicopper oxidase signatures | | Cu-oxidase Pfam domains | | | copper-binding motifs | | | | Comparison with the most homologous laccase reported | | | | |
| --- | --- | --- | --- | --- | --- | --- | --- | --- | --- | --- | --- | --- | --- | --- | --- | --- |
|  |  |  | **PS00079** | **PS00080** | **PF00394** | **PF07731** | **PF00394** | **Cu1** | **Cu2** | **Cu3** | **Cu4** | **Identity %** | **Query cover %** | **Protein Number accession** | **Organism** | **Reference** |
| g253.t1 | 553 | 43 | X | X | X | X | X | X | X | X |  | 57 | 89 | 2HRG_A | *Trametes trogii* | *(1), (2)* |
| g3223.t1 | 535 | 40 | X | X | X | X | X | X | X | X |  | 57 | 97 | 5MHW_A | *Steccherinum murashkinskyi* | *(3)* |
| g5839.t1 | 146 | 40 |  |  | X |  |  |  |  |  |  | 65 | 100 | 1GYC_A | *Trametes versicolor* | *(4), (5)* |
| g6228.t1 | 508 | 40 |  | X | X | X | X | X |  | X | X | 58 | 96 | 3KW7_A | *Trametes Sp. Ah28-2* | *(6)* |
| g6430.t1 | 576 | 40 | X | X | X | X | X | X |  |  |  | 50 | 90 | 1GYC_A | *Trametes versicolor* | *(4), (5)* |
| g8544.t1 | 696 | 43 | X | X | X | X | X | X | X |  |  | 58 | 72 | 1GYC_A | *Trametes versicolor* | *(4), (5)* |
| g12653.t1 | 545 | 43 | X | X | X | X | X | X | X | X |  | 46 | 99 | 1V10_A | *Rigidoporus lignosus* | *(7), (8)* |
| g12662.t1 | 529 | 40 | X | X | X | X | X | X | X | X |  | 57 | 96 | 5MHW_A | *Steccherinum murashkinskyi* | *(3)* |
| g16121.t1 | 580 | 43 | X | X | X | X | X | X | X | X |  | 52 | 88 | 1GYC_A | *Trametes versicolor* | *(4), (5)* |
| g16512.t1 | 558 | 40 | X |  | X | X | X | X | X | X |  | 58 | 87 | 1GYC_A | *Trametes versicolor* | *(4), (5)* |
| g16540.t1 | 540 | 43 | X | X | X | X | X | X | X | X | X | 61 | 96 | 3KW7_A | *Trametes Sp. Ah28-2* | *(6)* |
| g503.t1 | 733 | 40 |  |  | X | X | X | X |  |  |  | 27 | 86 | 3DKH_A | *Melanocarpus albomyces* | *(9)* |
| g1684.t1 | 531 | 40 | X | X | X | X | X |  |  | X |  | 37 | 93 | 4JHU_A | *Coriolopsis caperata* | *(10)* |
| g2824.t1 | 638 | 40 | X | X | X | X | X | X | X | X |  | 36 | 81 | 1ZPU_A | *Saccharomyces cerevisiae* | *(11)* |
| g3514.t1 | 249 | 21 |  | X |  |  |  |  |  |  |  | 29 | 40 | 3SQR_A | *Botrytis aclada* | *(12)* |

**
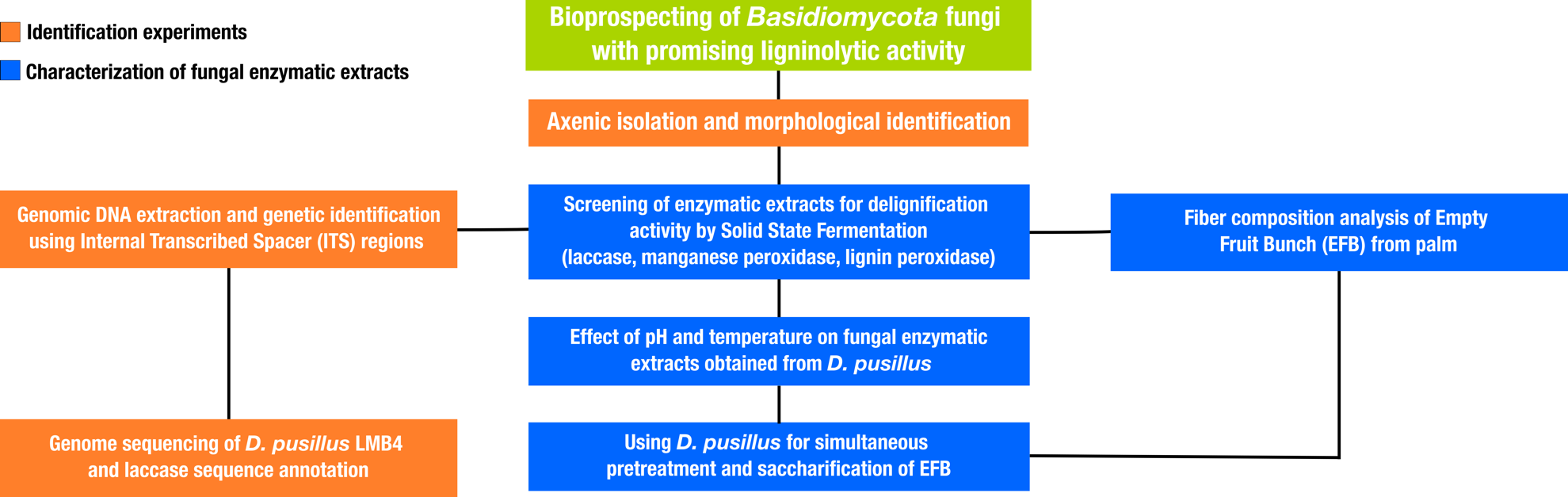
**

**Figure S1.** Overall strategy for bioprospecting, fungi isolation & identification, experimental characterization of ligninolytic activity, and genome sequencing performed in this study.

**References**

1. Ferraroni, M., Myasoedova, N. M., Schmatchenko, V., Leontievsky, A. A., Golovleva, L. A., Scozzafava, A., and Briganti, F. (2007) Crystal structure of a blue laccase from Lentinus tigrinus: evidences for intermediates in the molecular oxygen reductive splitting by multicopper oxidases. *BMC Structural Biology* **7**, 60-60

2. Matera, I., Gullotto, A., Tilli, S., Ferraroni, M., Scozzafava, A., and Briganti, F. (2008) Crystal structure of the blue multicopper oxidase from the white-rot fungus Trametes trogii complexed with p-toluate. *Inorganica Chimica Acta* **361**, 4129-4137

3. Polyakov, K. M., Gavryushov, S., Ivanova, S., Fedorova, T. V., Glazunova, O. A., Popov, A. N., and Koroleva, O. V. (2017) Structural study of the X-ray-induced enzymatic reduction of molecular oxygen to water by Steccherinum murashkinskyi laccase: insights into the reaction mechanism. *Acta Crystallographica Section D* **73**, 388-401

4. Antorini, M., Herpoël-Gimbert, I., Choinowski, T., Sigoillot, J.-C., Asther, M., Winterhalter, K., and Piontek, K. (2002) Purification, crystallisation and X-ray diffraction study of fully functional laccases from two ligninolytic fungi. *Biochimica et Biophysica Acta (BBA) - Protein Structure and Molecular Enzymology* **1594**, 109-114

5. Piontek, K., Antorini, M., and Choinowski, T. (2002) Crystal Structure of a Laccase from the FungusTrametes versicolor at 1.90-Å Resolution Containing a Full Complement of Coppers. *Journal of Biological Chemistry* **277**, 37663-37669

6. Ge, H., Gao, Y., Hong, Y., Zhang, M., Xiao, Y., Teng, M., and Niu, L. (2010) Structure of native laccase B from Trametes sp. AH28-2. *Acta Crystallographica Section F: Structural Biology and Crystallization Communications* **66**, 254-258

7. Cambria, M., Cambria, A., Ragusa, S., and Rizzarelli, E. (2000) Production, Purification, and Properties of an Extracellular Laccase from Rigidoporus lignosus. *Protein Expression and Purification* **18**, 141-147

8. Garavaglia, S., Teresa Cambria, M., Miglio, M., Ragusa, S., Iacobazzi, V., Palmieri, F., D'Ambrosio, C., Scaloni, A., and Rizzi, M. (2004) The Structure of Rigidoporus lignosus Laccase Containing a Full Complement of Copper Ions, Reveals an Asymmetrical Arrangement for the T3 Copper Pair. *Journal of Molecular Biology* **342**, 1519-1531

9. Hakulinen, N., Andberg, M., Kallio, J., Koivula, A., Kruus, K., and Rouvinen, J. (2008) A near atomic resolution structure of a Melanocarpus albomyces laccase. *Journal of Structural Biology* **162**, 29-39

10. Glazunova, O. A., Polyakov, K. M., Fedorova, T. V., Dorovatovskii, P. V., and Koroleva, O. V. (2015) Elucidation of the crystal structure of Coriolopsis caperata laccase: restoration of the structure and activity of the native enzyme from the T2-depleted form by copper ions. *Acta Crystallographica Section D* **71**, 854-861

11. Taylor, A. B., Stoj, C. S., Ziegler, L., Kosman, D. J., and Hart, P. J. (2005) The copper-iron connection in biology: Structure of the metallo-oxidase Fet3p. *Proceedings of the National Academy of Sciences of the United States of America* **102**, 15459-15464

12. Osipov, E., Polyakov, K., Kittl, R., Shleev, S., Dorovatovsky, P., Tikhonova, T., Hann, S., Ludwig, R., and Popov, V. (2014) Effect of the L499M mutation of the ascomycetous Botrytis aclada laccase on redox potential and catalytic properties. *Acta Crystallographica Section D: Biological Crystallography* **70**, 2913-2923
