# Supplementary material for: Genome sequencing and functional characterization of a Dictyopanus pusillus fungal enzymatic extract offers a promising alternative for lignocellulose pretreatment of oil palm residues
Source: PLoS One. 2020 Jul 30;15(7):e0227529. doi: 10.1371/journal.pone.0227529 (PMC7392265; doi:10.1371/journal.pone.0227529)
Supplement: Attachment — Submitted filename: Rueda Response To Reviewers.docx [file pone.0227529.s002.docx]

Montreal, May 30, 2020

Katherine A. Borkovich, Ph.D.

Academic Editor

PLOS ONE

**Re: Revision of manuscript ID FJ-18-0974**

Dear Prof. Borkovich,

Thank you for sending the reviews of the manuscript entitled *“Genome sequencing and functional characterization of a Dictyopanus pusillus fungal enzymatic extract offers a promising alternative for lignocellulose pretreatment of oil palm residues”* by Andrés M. Rueda, Yossef López de los Santos, Antony T. Vincent, Myriam Létourneau, Inés Hernández, Clara I. Sánchez, Daniel Molina V., Sonia A. Ospina, Frédéric J. Veyrier and Nicolas Doucet.

We have revised the manuscript based on suggestions and recommendations provided by the reviewers. We thank you and the reviewers for taking the time to provide helpful suggestions to make our manuscript stronger. We hope you and the reviewers will find our revised manuscript suitable for publication.

Best regards,

Nicolas Doucet, Ph.D.

Professor

**Comments from the Editor**

*1. Please provide an amended statement that declares *all* the funding or sources of support (whether external or internal to your organization) received during this study. (…) Please also include the statement “There was no additional external funding received for this study.” in your updated Funding Statement. Please include your amended Funding Statement within your cover letter. We will change the online submission form on your behalf.*

**Response**: Here is our updated Funding Statement:

This work was partially supported by a grant from Universidad Industrial de Santander, Vicerrectoria de Investigación y Extension (Grant number 5199) (to C.I.S. and D.M.V.), Industrias Acuña INAL LTDA (Grant number 8712) (to C.I.S.), and a Natural Sciences and Engineering Research Council of Canada (NSERC), via Discovery Grant RGPIN-2016-05557 (to N.D.). A.T.V. received a Postdoctoral Fellowship from NSERC. A.M.R. was supported by a doctoral scholarship from the Colombian Departamento Administrativo de Ciencia, Tecnología e Innovación (Colciencias) (PhD scholarship 567, 2012), and was the recipient of a scholarship from the Emerging Leaders in the Americas Program (ELAP) from the Government of Canada. F.V. and N.D. hold Fonds de Recherche Québec-Santé (FRQS) Research Scholar Junior 1 and Senior Career Awards, respectively (numbers 35038 and 281993). There was no additional external funding received for this study.

2. *Please provide an amended Competing Interests Statement that explicitly states this commercial funder. (…) Within this Competing Interests Statement, please confirm that this does not alter your adherence to all PLOS ONE policies on sharing data and materials by including the following statement: "This does not alter our adherence to PLOS ONE policies on sharing data and materials.” Please respond by return email with your amended Competing Interests Statement and we will change the online submission form on your behalf.*

**Response**: Competing Interests: Private funding provided by Industrias Acuña INAL LTDA (Grant number 8712) resulted from an internal competition by the Universidad Industrial de Santander (UIS) to support part of the current research project performed in the laboratory of Clara I. Sánchez at UIS (Colombia). Industrias Acuña INAL LTDA does not commercially benefit from the publication of this manuscript, nor was it involved in the conception, design, processing, analysis, or communication of the results and research project. All authors are fully committed to the full disclosure of all results and research materials resulting from the current research. All authors proclaim that this declaration of competing interests does not alter their adherence to all the PLOS ONE policies on sharing data and material.

*3. We note that you have included the phrase “data not shown” in your manuscript. Unfortunately, this does not meet our data sharing requirements. PLOS does not permit references to inaccessible data. We require that authors provide all relevant data within the paper, Supporting Information files, or in an acceptable, public repository. Please add a citation to support this phrase or upload the data that corresponds with these findings to a stable repository (such as Figshare or Dryad) and provide and URLs, DOIs, or accession numbers that may be used to access these data. Or, if the data are not a core part of the research being presented in your study, we ask that you remove the phrase that refers to these data.*

**Response**: These data were originally presented in a complementary fashion and were not essential for the understanding of the core research presented in this work. Consequently, we have removed the corresponding phrase.

*4. Please include captions for your Supporting Information files at the end of your manuscript, and update any in-text citations to match accordingly.*

**Response**: As requested, we have added captions for our Supporting Information files at the end of our manuscript. In-text citations were verified and correctly cited.

**Comments from Reviewer 1.**

*1. Throughout the text phrase “fungal enzymatic extract” is used in title it’s just fungal extract please check if the “fungal enzymatic extract” can be used.*

**Response**: We thank the reviewer for highlighting this discrepancy. For clarity and consistency, we have updated the text to include “fungal enzymatic extract” in all previously ambiguous instances that did not immediately relate to *D. pusillus* or any other fungi listed in each context, including in the title of the manuscript.

*2. In Page no 4 line “We found that laccase activity was the main enzymatic contributor to our fungal extracts, which included a highly active isolate from D. pusillus LMB4.” must be moved to the result section*

**Response**: Respectfully, we believe it is important for a reader to get a general idea of the main conclusions and results of the manuscript at the end of the introduction. We consider that this sentence is sufficiently broad and general to encompass this overall idea. We obviously discuss these data in more detail in the Results sections.

*3. Please elaborate the methodology for preparation of wheat bran extract*

**Response**: In response to this request, we have clarified and extended our methodological description of wheat brand extraction and fungi isolation in Section 2.1: “Isolation of the collected fungi was performed in wheat bran extract agar composed of 18 g.L^-1^ agar, 10 g.L^-1^ glucose, 5 g.L^-1^ peptone, 2 g.L^-1^ yeast extract, 0.1 g.L^-1^ KH_2_PO_4_, 0.1 g.L^-1^ MgSO_4_.7H_2_O, 0.085 g.L^-1^ MnSO_4_, 0.1 g.L^-1^ chloramphenicol, 0.1 g.L^-1^, 600 U.L^-1^ nystatin, and 1000 mL wheat bran extract. Wheat bran extract was obtained by filtering 175 g.L^-1^ of wheat brand soaked in distilled water for 1 h.”

*4. In Page 17-18, “These results are also in agreement with prior observations suggesting that basic pH is a desirable property for laccase used in biotechnological processes, since low pH values were linked to increased enzyme degradation.” The authors are suggesting basic pH has positive effect where the results shows otherwise. The enzyme is highly active in pH range of 3-4 during production (Fig 3A) and produced enzyme was highly stable in pH range of the 4 and 5 (Fig 3B). If possible the author must try higher pH then 5 such as 7-9 for claiming enzyme to be active in basic environment or must stick to the observations obtained and refrain from using active in basic environment or must stick to the observations obtained and refrain from using such statements contradicting their results.*

**Response**: This was a significant proofreading oversight on our part and we thank the reviewer for pointing out this contradiction in our analysis. We have removed these sentences, re-analyzed and re-written Section 3.4 relating to Figure 3 to more appropriately describe our observations, in addition to including quantifiable data and statistical analyses. We have also updated the quality and readability of Figures 3, 4 and 5.

*5. In Fig 6 components A, B, C, D, E were explained in figure title, but what does each image suggest is not represented. You have used small “a” and “b” in figure to denote both figure; however in the marking for table, it is denoted by capital A and B. Further capital A, B was used to represent different parameters. It is confusing for a reader, so please represent what does fig a) and b) or may use Fig 6 (i) or (ii)*

**Response**: We agree with the reviewer that panel numbering and identification was confusing in this figure. Consequently, we made a new version of this figure with renamed panels i) and ii). We also standardized and improved font usage and consistency in accordance with other figures.

**Comments from Reviewer 2.**

*1. The information on genome sequencing is scanty.*

**Response**: We apologize for the lack of precision in our original description of the genome sequencing and assembly methods. We have extended Sections 2.10 and 3.6 to clarify this, in addition to providing additional methodological and analyzing details.

*2. The statistical data/p values in Figures 3, 4, 5 are missing. (…) Information on statistical optimization cellulolytic digestion is not available.*

**Response**: We have clarified statistical data calculations in the legends of Figures 3, 4 and 5. We have also better outlined and described that the fungal enzymatic extracts exhibiting laccase activity and cellulases were subjected to a multilevel factorial experimental design (3 levels with 5 variables) to evaluate significant variables in the experimental process. This description is now better highlighted, cited and discussed in Sections 2.9 and 3.5. As requested, we also present all statistical p-values.

**Comments from Reviewer 3.**

*1. It would be better to have a figure to show the whole experiment design, to show the whole process of the study, including fungal strain isolation, sequencing, enzymes determination, biomass pretreatment and so on.*

**Response**: We thank the reviewer for this great suggestion. We have built a new experimental design figure that provides overall information on the strategy and procedures that were used in the current study. This is now Figure S1.

*2. There are a lot of uncommon abbreviations, such as EFB, SPS, very hard to following, it is better just write the full name.*

**Response**: As requested, we have removed all EFB and SPS abbreviations and replaced them with their respective full descriptions.

*3. In the page 19, the author said, the genome heterozygosity at 13,53%, please explain more about this heterozygosity, not every reader are familiar what this really means, it come from the sampling or it should be, because this fungus strain...*

**Response**: In addition to expanding our explanation of genome sequencing methodology and analysis, we have also clarified the genome heterozygosity meaning in Section 2.10 and discussion in Section 3.6.
